# The Rate and Spectrum of Spontaneous Mutations in *Mycobacterium smegmatis*, a Bacterium Naturally Devoid of the Postreplicative Mismatch Repair Pathway

**DOI:** 10.1534/g3.116.030130

**Published:** 2016-05-17

**Authors:** Sibel Kucukyildirim, Hongan Long, Way Sung, Samuel F. Miller, Thomas G. Doak, Michael Lynch

**Affiliations:** * Department of Biology, Indiana University, Bloomington, Indiana 47405; † Department of Biology, Hacettepe University, Ankara, 06800 Turkey; ‡ Department of Bioinformatics and Genomics, University of North Carolina, Charlotte, North Carolina, 28223; § National Center for Genome Analysis Support, Indiana University, Bloomington, Indiana 47405

**Keywords:** mutation accumulation, GC bias, *Mycobacteria*

## Abstract

*Mycobacterium smegmatis* is a bacterium that is naturally devoid of known postreplicative DNA mismatch repair (MMR) homologs, *mutS* and *mutL*, providing an opportunity to investigate how the mutation rate and spectrum has evolved in the absence of a highly conserved primary repair pathway. Mutation accumulation experiments of *M. smegmatis* yielded a base-substitution mutation rate of 5.27 × 10^−10^ per site per generation, or 0.0036 per genome per generation, which is surprisingly similar to the mutation rate in MMR-functional unicellular organisms. Transitions were found more frequently than transversions, with the A:T→G:C transition rate significantly higher than the G:C→A:T transition rate, opposite to what is observed in most studied bacteria. We also found that the transition-mutation rate of *M. smegmatis* is significantly lower than that of other naturally MMR-devoid or MMR-knockout organisms. Two possible candidates that could be responsible for maintaining high DNA fidelity in this MMR-deficient organism are the ancestral-like DNA polymerase DnaE1, which contains a highly efficient DNA proofreading histidinol phosphatase (PHP) domain, and/or the existence of a uracil-DNA glycosylase B (UdgB) homolog that might protect the GC-rich *M. smegmatis* genome against DNA damage arising from oxidation or deamination. Our results suggest that *M. smegmatis* has a noncanonical Dam (DNA adenine methylase) methylation system, with target motifs differing from those previously reported. The mutation features of *M. smegmatis* provide further evidence that genomes harbor alternative routes for improving replication fidelity, even in the absence of major repair pathways.

Spontaneous mutations play a central role in most evolutionary processes, and are responsible for nearly all forms of genetic disease. For this reason, it is important that we understand how the mutation rate and spectrum evolves across a wide range of organisms. Mutations arise from complex interactions between processes that damage DNA (exogenous and endogenous), prevent damage, and repair damage (Zhou and Elledge 2000), and, like most traits, the rate of mutation is determined by an interaction of the environment and these genetic factors. Previously, the mutation rate and spectrum has been studied by comparing putatively neutral sites in specific genes (Graur and Li 2000; Wielgoss *et al.* 2011), or by fluctuation tests using reporter-construct genes (Drake 1991). However, neither of these methods is free of potentially significant biases, because selection is likely to affect many putatively neutral sites and different genomic regions can have significantly different mutation rates (Hawk *et al.* 2005; Lynch 2007). By applying high-throughput sequencing technology to mutation-accumulation (MA) experiments, it is possible to generate an unbiased direct estimate of the genome-wide rate and spectrum of spontaneous mutations in an organism (Lynch *et al.* 2008; Halligan and Keightley 2009), allowing us to examine the forces driving the mutation process.

The general strategy of a bacterial MA experiment is to repeatedly bottleneck parallel lineages originated from a single cell for hundreds to thousands of generations. In this process, the strong bottlenecks minimize the efficacy of selection, enabling all but the most severely deleterious mutations to accumulate in an effectively neutral fashion (Muller 1927, 1928; Bateman 1959; Mukai 1964; Kibota and Lynch 1996). Through the MA process, unbiased estimates of genome-wide spontaneous mutation rates and spectra have been characterized for a number of eukaryotic and prokaryotic organisms (Lynch *et al.* 2008; Denver *et al.* 2009; Keightley *et al.* 2009, 2014, 2015; Ossowski *et al.* 2010; Lee *et al.* 2012; Sung *et al.* 2012a, 2012b; Behringer and Hall 2015; Dillon *et al.* 2015; Farlow *et al.* 2015; Long *et al.* 2015b; Ness *et al.* 2015), and have led to a general hypothesis explaining how mutation rates have evolved (Lynch 2010, 2011; Sung *et al.* 2012a). However, it remains unclear how DNA replication and repair interact to ultimately determine the mutation rate. Thus, further comparative work is needed to understand alternative evolutionary solutions to setting cellular mutation rates.

The *Mycobacterium* genus consists of a biologically diverse group of bacteria, 120 or more described species, including both human obligate pathogens, such as *M. tuberculosis* and *M. leprae*, and free-living saprophytes, such as *M. smegmatis* (Smith *et al.* 2009). *M. smegmatis* is relatively fast-growing, nonpathogenic, and genetically facile, so it provides an accessible model to study *Mycobacteria* in general (Snapper *et al.* 1990; Shiloh and DiGiuseppe Champion 2010). *M. smegmatis* has a genome of ∼7 Mb (Mohan *et al.* 2015), which is larger than most other *Mycobacterium* strains, including members of the pathogenic *M. tuberculosis* complex (MTB complex, ∼4.4 Mb), and *M. leprae* (∼3.3 Mb) (Brosch *et al.* 2001). The reduction in genome size of these mycobacterial pathogens is attributed to pathogenicity evolution (Brosch *et al.* 2001). *Mycobacteria* are classified as *Actinomycetales*, and some members of this group, such as *Nocardia* and *Corynebacterium*, have unusually high genomic GC-contents when compared to other bacteria (65.6%-GC in *M. smegmatis*). Genome sequencing has revealed that *Mycobacteria*, like all *Actinomycetales*, do not have any identifiable genes encoding the widely conserved mutLS-based postreplicative mismatch repair (MMR) system (Cole *et al.* 1998; Ford *et al.* 2013), suggesting that *Mycobacteria* lack canonical MMR, and thus might have unusual mutation features.

MMR maintains the fidelity of genomes by typically removing a fraction of the replication errors (Kunkel and Erie 2005; Lee *et al.* 2012). Previous studies showed that mutation rates of some MMR-knockout organisms are 10–100 × higher than in MMR-functional organisms (Lee *et al.* 2012; Lang *et al.* 2013). In addition, given the fact that MMR deficiency is common in some species (Garcia-Gonzales *et al.* 2012), an organism can significantly reduce DNA damage by using other repair or prevention pathways. Thus, because *M. smegmatis* is naturally devoid of known MMR genes, it may have compensatory mechanisms for efficient protection against mutations.

## Materials and Methods

### Mutation accumulation

Eighty independent *M. smegmatis* MC2 155 (ATCC 700084) MA lines were initiated from a single colony. 7H10 agar medium, with 0.5% glycerol and OADC enrichment 10% as recommended by ATCC, was used for the mutation-accumulation line transfers. Every 2 d, a single isolated colony from each MA line was transferred by streaking to a new plate, ensuring that each line regularly passed through a single-cell bottleneck (Kibota and Lynch 1996). Each line passed through ∼4900 cell divisions (Supplemental Material, Table S1). The bottlenecking procedure used for this experiment ensures that mutations accumulate in an effectively neutral fashion. MA lines were incubated at 37° under aerobic conditions. Frozen stocks of all lineages were prepared by growing a final colony per isolate in 1 ml 7H9 broth medium with 0.2% glycerol and ADC enrichment 10%, incubated overnight at 37°, and frozen in 20% glycerol at −80°.

### DNA extraction and sequencing

The 75 lines that survived through the end of MA were prepared for whole genome sequencing. DNA was extracted with the Wizard Genomic DNA Purification kit (Promega, Madison, WI). DNA libraries for Illumina HiSequation 2500 sequencing (insert size 300 bp) were constructed using the Nextera DNA Sample Preparation kit (Illumina, San Diego, CA). Paired-end 150-nt read sequencing of MA lines was done by the Hubbard Center for Genome Studies, University of New Hampshire, with an average sequencing depth of 126 × across all lines (Table S1).

### Mutation identification and analyses

A consensus approach for identifying fixed base substitutions and small-indels in the MA lines was modified from Sung *et al.* (2015). Briefly, paired-end reads from each MA line were mapped to the reference genome (GenBank accession number: NC_018289.1) using BWA 0.6.2 (Li and Durbin 2009), and read alignment and duplicate-read removal around indels was performed using GATK (McKenna *et al.* 2010; DePristo 2011). The output was parsed with SAMTOOLS (Li *et al.* 2009); mapped reads needed to pass filters for sequencing/PCR/mismapping errors; 26 lines were removed from the final analysis due to library construction failure or cross-line contamination (Table S1). Candidate mutations were called if they differed from the consensus sequence of all MA lines. Using the BAM and SAM formatted files from the BWA pipeline, BreakDancer 1.1.2 (Chen *et al.* 2009) and Pindel 0.2.4w (Ye *et al.* 2009) were also used to realign reads and identify small-indels. Both the consensus pipelines and the realignment programs support the final reported indels.

### Statistics and calculations

We used R v3.1.0 (R Development Core Team 2014) for all statistical tests and calculations; 95% Poisson confidence intervals were calculated using a χ^2^ estimation (Johnson and Kemp 1993).

### Data availability

Raw sequence reported in this study has been deposited in NCBI SRA (Bioproject No.: PRJNA320082; Study No.: SRP074205).

## Results

To estimate the mutation rate in *M. smegmatis* MC2 155, a mutation-accumulation experiment was carried out for 381 d (∼4900 generations) with 80 independent lineages, all derived from the same ancestral colony of *M. smegmatis*. Every 2 d, a single colony from each line was restreaked onto a fresh plate, minimizing the effective population size. We analyzed the mutation rate and spectrum across 49 MA lines that were successfully sequenced and not contaminated by other MA lines.

### Mutation rates

Across the 49 sequenced *M. smegmatis* MA lines (with an average of 6.77 Mb analyzable sequence per line, 97% of the total genome), we identified 856 base-substitution changes (Table S1 and Table S2), yielding an overall base-substitution mutation rate of 5.27 × 10^−10^ (SE = 1.93 × 10^−11^) per site per generation, or 0.0036 per genome per generation. Our analysis also reveals 207 short insertions and deletions 1–27 bps in length (141 insertions, and 66 deletions), yielding an insertion/deletion rate of 1.27 × 10^−10^ (SE = 1.08 × 10^−11^) per site per generation (Table S1 and Table S3). Although the insertion rate is 2.1 × greater than the deletion rate, the total size of all insertions is 206 bp while the deletions total 225 bp, resulting in a net loss of 19 bp in DNA sequence across all lines, consistent with the universal prokaryotic deletion bias hypothesis (Mira *et al.* 2001). Of the small indels, 78.74% occur in simple sequence repeats (SSRs), *e.g.*, homopolymer runs (Table S3), and these small-indels comprised 15.33% of all mutations.

Using the annotated *M. smegmatis* MC2 155 genome (NCBI accession: NC_018289.1), we identified the functional context of each base substitution (Table S2). Across the 49 lines, 716 of the 856 (83.64%) substitutions are in coding regions (90% of the genome represents coding regions), while the remaining 140 are found at noncoding sites (Table S2). To test for the absence of selection in our experiment, we asked whether the ratio of nonsynonymous to synonymous mutations is significantly different from the random expectation. Given the codon usage and the transition/transversion ratio (see below) in *M. smegmatis*, the expected ratio of nonsynonymous to synonymous mutations is 2.60, which is not significantly different from the observed ratio of 2.11 (486/230) (χ^2^ = 3.00, *P* > 0.01). Thus, selection does not appear to have had a significant influence on the distribution of mutations in this experiment.

### Comparison of mutation rates with various bacteria

Mutation rates in MMR-deficient genome backgrounds in several prokaryotic and eukaryotic organisms have been investigated by using whole-genome sequencing of mutation-accumulation lines. Most of these studies have found that MMR deficiency results in a >100-fold increase in the mutation rate compared to wild-type lines. In striking contrast, MMR-devoid *M. smegmatis* has a mutation rate comparable to that of other naturally MMR-proficient wild-type organisms (Table 1). These results suggest that *M. smegmatis* employs mechanisms that somehow compensate for the absence of MMR in order to reach the same mutation rate as other organisms that harbor the essential MMR enzymes.

**Table 1:** Mutation rates of MMR deficient bacteria (numbers are in 10^−10^ site per generation)

Organism	Transitions		Transversions				Overall Mutation Rate	Overall Mutation Rate of Wild-Type MA Lines	Reference
	A:T	G:C	A:T	G:C	A:T	G:C			
	↓	↓	↓	↓	↓	↓			
	G:C	A:T	T:A	T:A	C:G	C:G			
*B. subtilis (mutS****^–^****)*	280.17	375.54	6.86	2.30	4.76	4.02	331.00	3.28	Sung *et al.* (2015)
*D. radiodurans (mutL****^–^****)*	18.70	17.20	0.89	0	0.89	0.44	18.60	4.99	Long *et al.* (2015a)
*E. coli* (*mutL***^–^**)	389.09	152.43	4.77	3.41	3.41	1.02	275.00	2.66	Lee *et al.* (2012)
*M. florum*	13.20	165.94	4.06	93.30	3.05	47.30	98.00	—	Sung *et al.* (2012a)
*M. smegmatis*	3.95	2.76	0.27	1.58	2.10	0.43	5.27	—	This study
*Pseudomonas fluorescens (mutS****^–^****)*	284.45	191.00	1.82	5.12	1.72	2.18	234.00	–*^a^*	Long *et al.* (2015b)

a No whole-genome sequence data available for wild-type strain.

Previous studies have observed low levels of nucleotide diversity in *M. tuberculosis* and *M. leprae* populations (Sreevatsan *et al.* 1997; Monot *et al.* 2009), and have proposed that this is a result of recent population bottlenecks (Smith *et al.* 2009). However, low levels of nucleotide diversity can also be explained by low mutation rates (Lynch 2010). Low mutation rates observed in pathogenic strains of *M. tuberculosis* (2- to 7-fold lower than that observed in *M. smegmatis* in this study) are consistent with the latter explanation (Ford *et al.* 2011). However, the mutation rate difference between *M. smegmatis* and *M. tuberculosis* could also result from different experimental systems. Ford *et al.* (2011) used living infected macaques during latent infections to accumulate mutations, and detected only 14 base-substitution mutations, but no A:T→T:A or A:T→C:G transversions. The *in vivo* environment could have biased the mutations by strong selection such as the host immune system, even if high numbers of mutations had been detected. Thus, it cannot be confirmed that *M. tuberculosis* has a similar mutation spectrum with *M. smegmatis* by comparing our data with *M. tuberculosis* mutations detected from whole-genome sequencing in Ford *et al.* (2011). But, a mutation accumulation experiment using *M. tuberculosis* may provide a clear answer to this.

### Mutation spectrum

Across the 49 MA lines, we found 511 transitions and 345 transversions, resulting in a transition/transversion ratio of 1.48. Among the base-substitution changes, there are 302 G:C→A:T transitions and 173 G:C→T:A transversions at GC sites, yielding a mutation rate in the AT direction of ${\text{µ}}_{\text{G/C}\to \text{A/T}}=$ 4.34 × 10^−10^ per site per generation. In contrast, 209 A:T→G:C transitions and 111 A:T→C:G transversions yielded a mutation rate in the G:C direction of ${\text{µ}}_{\text{A/T}\to \text{G/C}}$ = 6.04 × 10^−10^ per site per generation (Table S1), which is significantly higher than the ${\text{µ}}_{\text{G/C}\to \text{A/T}}$ rate (95% Poisson confidence intervals for ${\text{µ}}_{\text{G/C}\to \text{A/T}}$3.96−4.74 × 10^−10^, for ${\text{µ}}_{\text{A/T}\to \text{G/C}}\;$5.40−6.75 × 10^−10^). Given these conditional A/T↔G/C mutation rates, the expected GC content from mutation alone is 58.2% (SE = 4.67%), significantly lower than the actual chromosomal GC content of 65.6%.

Methylated bases are mutational hotspots in bacteria (Schaaper and Dunn 1991; Lee *et al.* 2012). Previous studies have found that mycobacterial species contain methyltransferases that are not canonical Dam or Dcm DNA methyltransferases (Shell *et al.* 2013; Sharma *et al.* 2015; Zhu *et al.* 2016), but are associated with the presence of 6-methyladenine in their genomes (Shell *et al.* 2013). We examined mutation rates at noncanonical Dam target sites (Schlagman and Hattman 1989; Clark *et al.* 2012; Shell *et al.* 2013), and previously suggested noncanonical methylation sites in other bacteria (Long *et al.* 2015a), and found that 45% of the A:T→C:G transversions (50 of 111) fall in motifs of 5′GACC3′ (30) and 5′CACC3′ (20), a 6.8-fold elevation from the transversion rate of A:T sites not falling in these motifs. The mutation hotspots at noncanonical Dam target sites suggest methylation at these sites (Table S4). Surprisingly, the reported Mycobacterial Adenine Methyltransferase sites 5′GAATTC3′ (Nikolaskaya *et al.* 1985) and 5′CTGGAG3′ (Shell *et al.* 2013) are not enriched for A:T→C:G transversions, suggesting that these sites are not routinely methylated in the *M. smegmatis* genome.

Although the presence of 5-methylcytosines was previously reported in *M. tuberculosis* and *M. smegmatis* genomes (Srivastava *et al.* 1981; Hemavathy and Nagaraja 1995), recent studies have found no 5-methylcytosine modification in the genomes of *M. tuberculosis* complex strains (Shell *et al.* 2013; Zhu *et al.* 2016). In our study, 21% (65 of 302) of the G:C→A:T transitions fall in the motifs of 5′CCGC3′, 5′CGCC3′, 5′CGCG3′, and 5′CGGC3′, which were not reported previously, and 48% (145 of 302) fall in 5′CpG3′ sites, ∼3.5-fold elevated from cytosines not in these sites (Table S5). As shown in yeasts, cytosines at 5′CpG3′ sites may have an elevated mutation rate even without methylation (Zhu *et al.* 2014; Behringer and Hall 2015; Farlow *et al.* 2015).

## Discussion

Because most mutations have slightly deleterious fitness effects (Baer *et al.* 2007; Eyre-Walker and Keightley 2007), natural selection is thought to operate to minimize replication errors and maximize DNA repair efficiency (Kimura 2009). It has been proposed that the efficacy of selection in reducing mutation rates is determined by the power of random genetic drift, which is inversely proportional to the effective population size (Lynch 2010, Lynch 2011). Given this theoretical framework, because population sizes in free-living bacteria are expected to be large (on the order of 10^7^–10^9^), we expect different species to have roughly similar per genome mutation rates if they have similar population sizes (Sung *et al.* 2012a; Sniegowski and Raynes 2013). Consistent with this idea, *M. smegmatis* has roughly the same mutation rate as other free-living bacteria (Lee *et al.* 2012; Long *et al.* 2015b; Sung *et al.* 2015). Yet *M. smegmatis* lacks critical MMR enzymes, suggesting that either *Mycobacterium* pre-MMR replication fidelity is higher than that of other prokaryotes, or that alternative biochemical mechanisms are used to arrive at the equivalent mutation rates.

### Alternative pathways for replication fidelity

Three main processes influence DNA-replication fidelity: nucleotide insertion fidelity of the DNA polymerase, removal of mispaired nucleotides by the DNA proofreading exonuclease, and MMR. Sequential action of these three steps is responsible for the typically low bacterial error rate of ∼10^−10^ per base replicated (Schaaper 1993; Kunkel 2004). However, it remains possible that a deficiency in any one of these processes may be compensated for by increased fidelity in the others (Lynch 2012): in *M. smegmatis*, as in the case of *D*einococcus *radiodurans* (Long *et al.* 2015a), it appears that a mechanism arising from such evolutionary layering must compensate for MMR deficiency.

Replication of the *Escherichia coli* chromosome is performed by the DNA polymerase III holoenzyme, which replicates the leading and lagging strands simultaneously (Kelman and O’Donnell 1995). The alpha (α) and epsilon (ɛ) subunits have a major effect on fidelity of the DNA polymerase III holoenzyme, allowing DNA synthesis to proceed with ∼10^−7^ errors/bp replicated (prior to proofreading) (Schaaper 1993; Kunkel and Erie 2005). The proofreading subunit of the DNA polymerase, the epsilon (ε) exonuclease, is also essential for high-fidelity DNA replication in *E. coli*, with inactivation increasing the mutation rate up to 200-fold (Schaaper 1993). However, surprisingly, Rock *et al.* (2015) found that although the proofreading exonuclease in *M. tuberculosis* is present, it is completely dispensable for fidelity, and an alternative exonuclease contributes to replicative fidelity in *Mycobacteria*. They found that the *Mycobacterial* DNA polymerase DnaE1 performs DNA proofreading with a polymerase and histidinol phosphatase (PHP) domain; inactivation of the PHP domain increased the mutation rate by more than 3000-fold (Rock *et al.* 2015). This decrease in proofreading fidelity suggests that the burden of DNA repair placed on MMR in other species may instead be placed onto the DnaE1 proofreader in *Mycobacteria*.

### Role of DNA methylation in biased mutation spectrum

In the *M. smegmatis* genome a subset of adenine and cytosine sites have an elevated mutation rate. These sites are associated with specific sequence motifs: 45% of the A:T→C:G transversions (50 of 111) occur at adenines in the motifs 5′GACC3′ (30) and 5′CACC3′ (20), which are known noncanonical Dam methylation sites. In addition, 21% (65 of 302) of the G:C→A:T transitions occur at cytosines in the motifs 5′CCGC3′, 5′CGCC3′, 5′CGCG3′, and 5′CGGC3′, and overall 48% (145 of 302) of the G:C→A:T transitions fall in 5′CpG3′ sites. These two classes of mutation account for nearly a quarter of all base-substitution changes that we observed, and on balance they sum to a strongly biased G:C→A:T transition, making the overall A:T→C:G rate dependent on the mutation spectrum at unmethylated sites. While adenine methylation has been reported in *M. smegmatis*, cytosine methylation has not been seen (Shell *et al.* 2013; Zhu *et al.* 2016), although our results suggest this should be reexamined.

### Alternative forms of DNA repair

Different DNA repair processes may generate the unusual mutation spectrum observed in *M. smegmatis*. A near universal mutation bias toward A/T has been observed in most species (Hershberg and Petrov 2010), but we find a bias to G/C in *M. smegmatis*. Notably, *M. smegmatis* is GC-rich, suggesting that mutation bias may have a role in determining the GC content in this genome. For the species in which a GC mutation bias observed (Dillon *et al.* 2015; Long *et al.* 2015a), this may be a product of methylation, deamination, and/or repair. For example, *Mycobacteria* have a high level of redundancy in the base excision repair (BER) pathway (van der Veen and Tang 2015), which could reduce the number of G:C→T:A transversions (Wallace 2002) associated with oxidative damage (David *et al.* 2007). In both *Bacillus subtilis* and *E. coli*, MutY can compensate for MMR enzymes, by removing adenines that are mispaired with cytosines, and preventing G:C→A:T mutations (Kim *et al.* 2003; Bai and Lu 2007; Debora *et al.* 2011).

GC-rich genomes may deploy additional enzymes to survey the fidelity of GC-sites, which are highly susceptible to cytosine deamination (Dos Vultos *et al.* 2009).The UdgB enzyme plays a more important role in removing uracils in *M. smegmatis* than for bacteria with known MMR activities (Wanner *et al.* 2009; Malshetty *et al.* 2010). For example, based on conserved sequences, six Udg families have been identified in various eubacteria, with different substrate specificities (Pearl 2000; Sartori *et al.* 2002; Srinath *et al.* 2007; Lee *et al.* 2011). *Mycobacteria* encode one family 1 Ung, and one family 5 UdgB (Sartori *et al.* 2002). The latter has only been characterized in a few organisms such as hyperthermophilic archaea and *M. tuberculosis*, and eukaryotes do not have this enzyme (Sartori *et al.* 2002; Starkuviene and Fritz 2002; Hoseki *et al.* 2003; Srinath *et al.* 2007). *In vitro* assays show that UdgB removes uracil from both ssDNA and dsDNA (Sartori *et al.* 2002), and excises hypoxanthine (Hx) from oligonucleotide substrates *in vitro* (Sartori *et al.* 2002; Srinath *et al.* 2007). Wanner *et al.* (2009) found that the mutation frequency in a *udg* knockout strain of *M. smegmatis* is ∼8-fold higher relative to wild type (Wanner *et al.* 2009). Furthermore, *M. smegmatis* Ung is more efficient at excising uracils from hairpin-loop substrates than that of *E. coli* (Purnapatre and Varshney 1998), and the frequency of mutations in double *udgB ung* mutants is 56-fold higher than in wild-type *M. smegmatis* (Wanner *et al.* 2009). Similar to these results, Malshetty *et al.* (2010) showed synergistic effects of UdgB and Ung in mutation prevention in *M. smegmatis*: the mutation rate of a *udgB* knockout is ∼2.1-fold higher, and the rate of a *ung* knockout is ∼8.4-fold higher than wild-type *M. smegmatis*. But the double knockout (*udgB^–^ung*^–^) shows a ∼19.6-fold increase in mutation rate (Malshetty *et al.* 2010). By contrast, uracil DNA glycosylase (*ung*) mutants increase mutation frequency by only ∼2-fold in *B. subtilis* (López-Olmos *et al.* 2012), and ∼5-fold in *E. coli* (Duncan and Weiss 1982).

In conclusion, we have shown that *M. smegmatis* has a typical bacterial mutation rate, even though it lacks the near-universal MMR system, has an unusual A:T→C:G biased mutation spectrum, and has motifs for both probable adenine and cytosine methylation, which act as genomic mutational hotspots. We have discussed possible mechanisms that allow *M. smegmatis* to evolve a low mutation rate despite the apparent absence of MMR. Consistent with the drift-barrier hypothesis (Lynch 2010; Sung *et al.* 2012a), *M. smegmatis* has evolved to a summed replication fidelity and repair rate equal to that observed in most free-living bacteria. However, the lack of MMR in *M. smegmatis* necessitates compensatory selection to improve alternative enzymatic pathways that limit the mutation rate expected for its population size. Further biochemical assays are required to determine whether the discussed pathways replace the role of MMR with DNA replication fidelity, or if novel repair pathways exist.

## Supplementary Material

Supplemental Material
