# Supplementary material for: The Rate and Spectrum of Spontaneous Mutations in Mycobacterium smegmatis, a Bacterium Naturally Devoid of the Postreplicative Mismatch Repair Pathway
Source: G3 (Bethesda). 2016 May 17;6(7):2157–63. doi: 10.1534/g3.116.030130 (PMC4938668; doi:10.1534/g3.116.030130)
Supplement: Supplemental Material [file supp_6_7_2157__index.html]

The Rate and Spectrum of Spontaneous Mutations in Mycobacterium smegmatis, a Bacterium Naturally Devoid of the Postreplicative Mismatch Repair Pathway — Supplemental Material 

# The Rate and Spectrum of Spontaneous Mutations in *Mycobacterium smegmatis*, a Bacterium Naturally Devoid of the Postreplicative Mismatch Repair Pathway

## Supplemental Material for Kucukyildirim, *et al*, 2016

**Files in this Data Supplement:**

- Table S1 - *Mycobacterium smegmatis* base substitution and inserion-deletion (indel) summary statistics. (.xlsx, 60 KB)
- Table S2 - Base substitution details. (.xlsx, 108 KB)
- Table S3 - Indel details. (.xlsx, 46 KB)
- Table S4 - Potential Dam\* methylation site mutations. (.xlsx, 31 KB)
- Table S5 - Potential Dcm\* methylation site mutations. (.xlsx, 31 KB)
